# Utilizing the “teach-back” method to improve surgical informed consent and shared decision-making: a review

**DOI:** 10.1186/s13037-022-00322-z

**Published:** 2022-03-05

**Authors:** Kevin D. Seely, Jordan A. Higgs, Andrew Nigh

**Affiliations:** 1 College of Osteopathic Medicine, Rocky Vista University, Ivins, UT 84738 USA; 2 Division of Clinical Medicine and Surgery, Rocky Vista University, UT 84738 Ivins, USA

**Keywords:** Surgery, Preoperative education, Informed consent, Teach-back method, Shared decision-making

## Abstract

The teach-back method is a valuable communication tool that can be employed to improve patient safety and shared decision-making. Its utility in patient care has been studied extensively in many areas of clinical medicine. However, the literature on the use of teach-back in surgical patient education and informed consent is limited. Additionally, there is some ambiguity about the functional definition and performance of the teach-back method in the literature, consequently rendering this valuable tool an enigma. This review examines the current standards and ethics of preoperative informed consent and provides a concise, actionable definition of teach-back. The manner in which teach-back has been implemented in medicine and surgery is then examined in detail. Studies analyzing the use of teach-back in medicine have demonstrated its effectiveness and benefit to patient care. Further study on the use of teach-back to improve preoperative informed consent is supported by the few preliminary trials showing a positive effect after implementing the teach-back method in critical patient interactions.

## Introduction

Patient autonomy, shared decision-making, and informed consent are foundational components of ethical patient care [1]. Modern best practices concerning patient education and shared decision-making place responsibility on patients to participate in the healthcare-related decision-making process [2]. Therefore, practical interpersonal strategies are needed to support patients’ understanding of complex health information that enables them to make informed, high-consequence decisions. The teach-back method has been shown to aid in the shared-decision making process and improve health literacy and outcomes [3]. However, despite the proven benefits in patient education and outcomes in many areas of medicine, the literature on teach-back in the surgical literature is limited.

This review examines the literature and key findings pertaining to the teach-back method in medicine that can be implemented in surgical practice and pre-operative education. Because there is some ambiguity about the functional definition and performance of the teach-back method in the literature, a concise and actionable definition of teach-back is established. The evidence regarding the use of teach-back in medicine, including how teach-back is delivered, the effectiveness of teach-back across different healthcare settings and populations, and how teach-back might be applied to preoperative informed consent, is examined.

Communication is crucial to the delivery of patient-centered healthcare [4]. With the transition from a system and culture of medical paternalism to one of shared decision-making and patient autonomy has come the unprecedented importance of effective communication. Adverse health outcomes, compromised safety, and increased economic burden are attributable to communication gaps and the breakdown of physician-patient relationships [4, 5]. This is particularly true when considering pre-operative education and informed consent. Many factors contribute to effective communication, including tone, subject matter, patient anxiety, fear levels, patient expectations, physician workloads, fear of litigation, and concern of physical or verbal abuse [6]. The ultimate goals of physician-patient communication include mutual understanding, establishing a rapport, facilitating the exchange of information, and including patients in decision making [7].

Physicians have a legal and ethical responsibility to provide patients with information so that they can process the information and make appropriate decisions during the informed consent process [8]. An educated patient benefits the physician, both in terms of cooperation in the planned intervention and in reducing acrimony if complications arise [8]. The extent to which surgeons choose to educate patients preoperatively is inconsistent with no established norm. Notwithstanding the wide variety in delivery in terms of time length, place, and pace, informed consent is ethically and legally required prior to invasive medical and surgical procedures. At the very least, providers are legally required to provide consent forms to the patient, which they must sign prior to surgery. However, it is becoming increasingly evident that basic consent forms provide insufficient information to adequately guide the general population in decision-making. Manta et al. in a 2021 study on patient perspectives about informed consent, showed that consent forms are too complex to achieve appropriate patient comprehension [9]. In a 2014 letter to the editor, Bracaglia et al. argued that a conventional informed consent form does not achieve effective communication or comprehensive knowledge on the part of the patient at the time of signing. Furthermore, they assert that a patient’s signed permission does not assure that the patient fully comprehends its contents. They conclude that in a medical culture pursuant to increasing patient autonomy and self-determination, the first step toward a solution would be to assess the patients’ degree of understanding of the information presented [10].

**Fig. 1:**
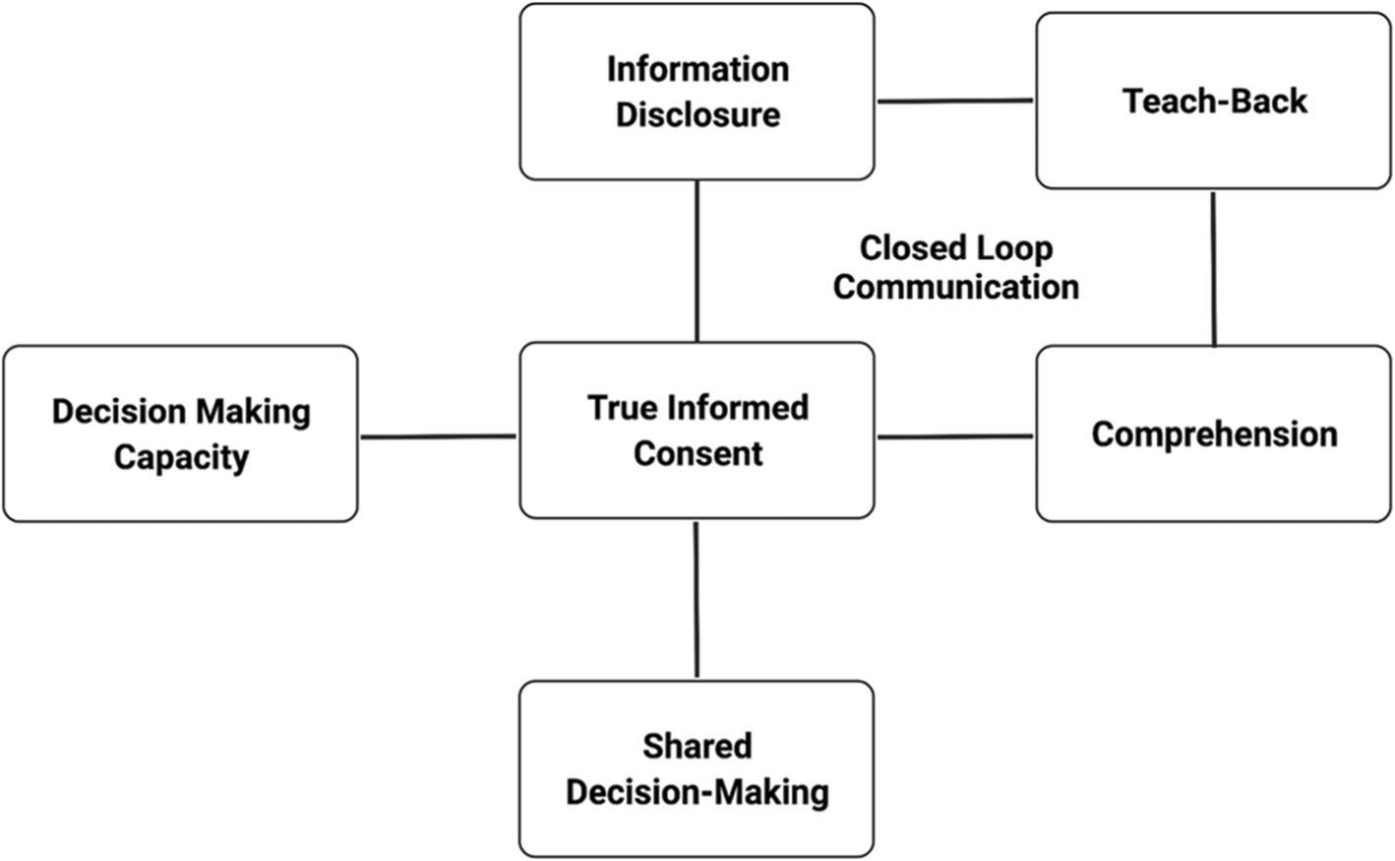
Teach-back adds a closed-loop communication aspect to the informed consent process. It facilitates enhanced informed consent and aids in the shared decision-making process

Informed consent occurs when patient-clinician communication results in a patient’s authorization to undergo a specific medical intervention [11]. The process should ideally help ensure both adequate disclosure on the part of the physician and sufficient comprehension on the part of the patient [12]. Consent is valid only when the patient has the capacity to consent, has discussed and understood all relevant information, consents voluntarily, and communicates their decision [13]. The process of informed consent can be described as having five core components: (1) assessment of decision-making capacity, (2) discussion of pertinent information, (3) assessing comprehension, (4) ensuring voluntary consent through collaborative deliberation, (5) formally obtaining consent with correct documentation and signature [14]. Teach-back has been proposed as an efficient way to improve the second and third components, assessing comprehension and aiding in the shared decision-making process (Fig. 1).

### Teach-back method

The teach-back method, a dynamic, interactive process, was created to improve the communication between provider and patient, allowing the physician to identify and resolve any misunderstandings in real-time, thus improving the comprehension of information [15, 16]. This technique may reveal cognitive, cultural, language, or health literacy barriers that increase the risk of miscommunication or unintended messaging [17]. As there is no standardized operational definition of feedback, the exact process used throughout the medical system may vary [18].

Teach-back consists of multiple steps involving the clinician introducing new information, assessing the recall of the patient by asking them to repeat what they understood, and by clarifying and tailoring the information to the patient’s level of understanding, the clinician will then reassess the patient’s understanding. It has been suggested that this cycle be repeated as many times as necessary for comprehension by the patient [19–21].

**Fig. 2:**
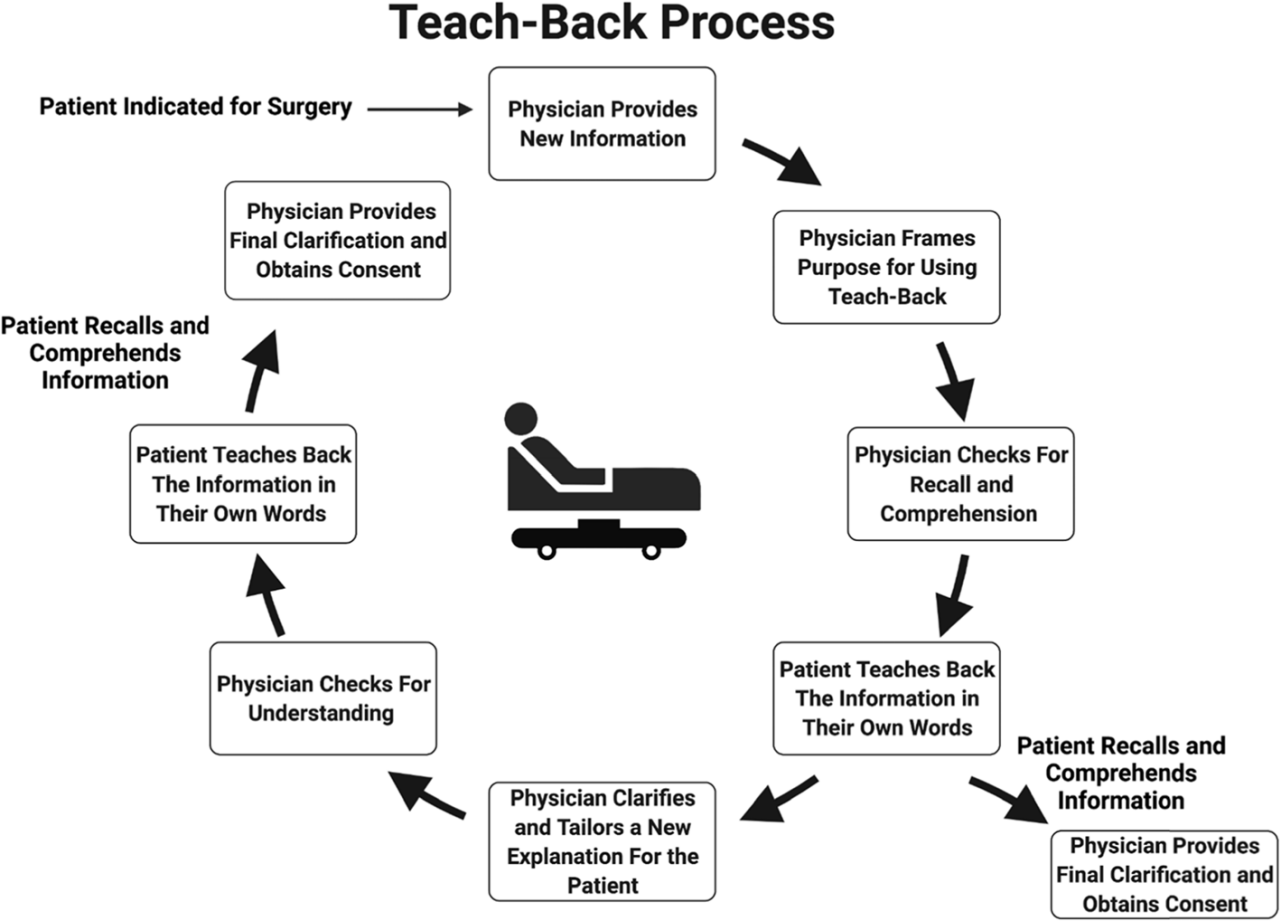
Teach-back is a dynamic, interactive, and patient-centered process that may require multiple repeated sequential explanations, checks for comprehension, and clarifications

The operative definition used in this paper consists of providing new information with several checks for recall; however, the initial check for recall must be preceded by a “framing statement.” The purpose of this statement is to place the focus of the conversation on effective communication between the patient and provider and may serve to reduce stigma experienced by the patient [18, 22]. An example of a framing statement would be “I want to make sure I explained correctly.” The framing statement should effectively place the focus of the teach-back process on the communication effectiveness of the physician and not the patient’s ability to understand. If during the process of teach-back, the patient successfully verbalizes adequate understanding of the subject matter, the process is complete (Fig. 2).

Despite the teach-back process having been shown to initiate more desirable outcomes for patients regardless of differences in age and education levels, it may not be utilized often enough in medical care [23]. Multiple studies have analyzed factors such as the use of medical jargon, assessment of patient understanding, and use of teach-back. These studies have shown that residents who are asked to evaluate the utilization of their communication with patients, tend to overestimate the effectiveness of their communication [24, 25]. The variance of communication effectiveness between provider and patient may contribute to problems that arise at the time of a patient’s care. Issues such as insufficient comprehension of one’s own medical diagnosis, procedural complications, or post-op care, may be tied to issues of physician communication.

### Ethical basis of informed consent

Truly informed consent protects the patient’s right to self-determination [8]. Respect for autonomy in the form of freedom to decide what happens to one’s own body is the key ethical rationale for gaining informed permission from patients. Included in this definition is the ability to accept or reject therapies that clinicians deem medically justified. In some cases, the need for consent may be outweighed by the need for urgent intervention. In emergency situations, the patient may be less capable of receiving, interpreting, or communicating information [26]. When possible, the medical facts of the case should be explained to the patient and with the patient’s permission to the family. In the case of language barriers between patient and provider, a translator trained in medical terminology is often required to transmit accurate information [27]. The ethics of surrogate decision-making is outside of the scope of this paper, and the authors direct the reader to a recent review written by Kim et al. addressing current issues surrounding surrogate decision-making and informed consent [28].

### Evolution of informed consent before surgery over time

Informed consent has evolved slowly. Early documentation of the patient-physician relationship was from the pages of the ancient Greek Corpus Hippocraticum. Although these papers did not describe the physician-patient relationship that we know today, they were among the first to describe the principles of beneficence and nonmaleficence, ethical standards for all physicians. The idea that physicians have a moral and ethical contract to the patients who enter into their care has evolved through the ideas of physicians such as French surgeon Henri de Mondeville, moralist John Gregory, Declaration of Independence signer Benjamin Rush, and Thomas Percival in his treatise on medical ethics [29]. These contributions led to the eventual publication of the “American Medical Association (AMA). Medical Ethics” in 1847, which outlined the honorable behavior of physicians. However, it was not until Worthington Hooker published a commentary on the AMA medical ethics code denouncing lying to patients, performing unnecessary procedures, and observing “the science of patient getting, to the neglect, to some extent, at least, of the science of patient curing” that honorable treatment of patients was emphasized [30].

Events of the early twentieth century laid the foundation for informed consent that we currently recognize. Informed consent developed through the contributions of many individuals with the outcome of increasing the capacity of physicians to serve the needs of their patients. It has enabled patients greater control over their own care and access to information that will allow them to make well-advised healthcare decisions. This stepwise process was developed through trial and error based on principles of battery and negligence [29]. Precedents of judicial cases led to the development of the modern standard of informed consent. Summaries of these key cases and events are provided in Table 1.

**Table 1:** Legal developments in informed consent in the twentieth century

Date	Case	Significance	References
1905	Mohr v. Williams	When entering into a contract, the physician can operate to the extent of the consent given, but no further.	[31]
1906	Pratt v. Davis	Limited implied consent to emergencies or when the patient knows the consequences of allowing the physician to exercise professional judgment	[32]
1913	Rolater v. Strain	Strengthened the patient’s control over their care	[33]
1914	Schloendorff v. Society of NY Hospital	Competent individuals have a right to decide what will be done to their bodies. Performing surgery without a patient’s consent is assault, and the surgeon may be held liable	[34]
1957	Salgo v. Stanford	Physicians must disclose facts necessary to make an intelligent consent for the proposed treatment	[35]
1960	Natanson v. Kline	If injury results from a known risk that is not disclosed to the patient, the physician may be liable	[36]
1972	Cobbs v. Grant and Wilkinson v. Vesey	Whether a patient should proceed with therapy requires reference to the values of that patient and thus are not exclusively medical determinations	[37, 38]
1973	Legislation	Patient’s Bill of Rights published	
1975-1977	Legislation	25 states enacted informed consent laws to decrease malpractice suits.	
1980	Truman v. Thomas	Physicians must apprise the patient of the risks of not undergoing treatment	[39]

### Current standards and requirements

Assessment of decision-making capacity is based on a determination that the patient is competent, meaning, the patient is able to comprehend their medical problems and make decisions for their own health care [40]. When competency has been ascertained, the physician is obligated to present to the patient pertinent information that enables them to make a well-informed decision. Information provided should consist of their diagnosis, proposed treatment or procedure with its accompanying risks and benefits, alternative treatment options with respective risks and benefits, additional procedures that may become necessary during the course of a procedure, and the risks of refusing treatment. Difficulty may arise in evaluating how much information is pertinent to the patient with regards to a patient’s health education and background.

Laws in some states detail the information that must be presented for a specific procedure, but ultimately, the depth of the discussion is left to the discretion of the physician [37, 41]. The physician is also obligated to disclose information truthfully when asked by the patient, including the number of similar procedures performed and success rates. Failure to do so would leave the physician open to allegations of fraud and misrepresentation and “negligent nondisclosure” [42, 43]. Additionally, any financial conflicts of interest, such as an instance of a provider having part ownership of a lab or facility, must be disclosed and as well as any commercial interest in the patient’s cells, devices, or techniques [44]. The patient has the ultimate right to be advised about any individuals participating in their care. This includes but is not limited to attendings, residents, or students [45].

Physicians may proceed with the informed consent process in a variety of ways chosen to best address a particular patient’s needs. Informed consent can be discussed verbally with the use of educational material or interpreters. Providing the patient with relevant materials is especially beneficial in helping patients retain more information and achieve a better understanding of procedures. Patients additionally feel more involved, have a better sense of their values, and are better able to become more involved in their healthcare [46–48]. Because comprehension of informed consent has been shown to depend on the educational level of the patient [49], physicians may utilize any number of supplemental materials or other interventions to cater to an individual patient’s needs.

Because many medical procedures are complicated and often multifaceted, it is critically important for physicians to document the informed consent process. Patients may have difficulty.

remembering all the facts of their procedure, so the discussion must be documented to avoid any misunderstanding or litigation [50, 51]. The physician in charge of the procedure should take responsibility for ensuring that the informed consent is presented and properly documented [52, 53]. In the documentation, they should be sure to include what was discussed in the patient visit, who was in attendance, and the other elements of well-informed consent to ensure a thorough report.

### Teach-Back in medicine

Teach-back has been shown to be a valuable strategy that can improve the safety and quality of health care and improve health literacy [54], which has been adopted in a variety of fields, to varying degrees of consistency. A 2019 study on the use of teach-back by medical residents revealed that residents believe they are using teach-back to confirm patient understanding 60% of the time when they actually used teach-back only 2.5% of the time. Following the educational intervention, the residents used teach-back 53% of the time. As a result, was found that teach-back language was collaborative and patient-centered, and all but two of 78 patients confirmed their medication and discharge plan after teach-back intervention [24].

Informed consent regarding heart medication instruction is one area in which teach-back has been successfully implemented. White et al. conducted a prospective cohort study in 2013, that involved 276 heart-failure patients over 13 months. Patients were educated and evaluated using the teach-back method in addition to collaborative care planning and patient education. Data on ability to recall educational information while hospitalized and during follow-up, approximately seven days after hospital discharge were collected and compared to readmissions data. Data analysis showed that the teach-back method is an effective method used to educate and assess learning and that patients educated longer retained significantly more information than did patients with shorter instruction. The study did not show a correlation between patient knowledge and a decrease in readmission [55]. Another study of the effect of teach-back on knowledge, outcome, readmission, and quality of life in heart-failure patients showed significant improvements in patients’ knowledge and performance immediately after teach-back education, although they found that this effect was lessened as time from discharge increased. No correlation between teach-back and decreased frequency of readmission was uncovered [56]. This study also showed a statistically significant increase in patient-perceived quality of life through teach-back education in terms of vitality, general health, and social functioning.

One meta-analysis analyzing the use of teach-back in the management of chronic disease found that teach-back showed positive effects in a wide range of healthcare outcomes, including improved disease-specific knowledge, adherence to medication regimens and diet modifications and foot care [21]. Another meta-analysis published in 2017 by Yen et al. came to similar conclusions noting that the use of the teach-back method is effective in reinforcing or confirming patient understanding. Additional findings of this analysis showed that none of the studies reported harmful outcomes and that the teach-back method, therefore, can be safely used to increase patient understanding and satisfaction [57].

Teach-back has also been shown to be effective in educating patient home caregivers. A significant increase in recall of the purpose and side-effects of new medications was shown in a 2019 study by Prochnow et al. in which 25 registered nurses and 74 patients with some of their caregivers were observed in instruction sessions and surveyed following the discussion about medication importance, adherence, and side-effects. In a quality improvement project aiming to improve caregivers’ confidence in caring for hospice patients and decreasing hospitalizations, a pre-test-post-test model was used to analyze the effectiveness of teach-back in reducing hospitalizations in hospice patients. After the intervention, the teach-back group had zero hospitalizations compared with two hospitalizations for the non-teach-back group. Patient-caregiver “confidence” increased from 58% to 81%, pre- to post-intervention. These authors concluded that teach-back is a cost-effective teaching methodology that can be implemented by any discipline to improve patient-provider communication and patient outcomes [58].

Teach-back has also been widely studied in oncology. A systematic review conducted by Choi et al. found 246 published articles pertaining to the use of the teach-back method with cancer patients. The study found that teach-back interventions promoted positive health outcomes, including increased happiness, decreased uncertainty, better self-efficacy and self-management behavior, lessened symptom experience, diminished distress and anxiety, and improved health literacy among cancer patients. Whether or not these same outcomes translate to the surgical care setting has not been studied extensively [59].

Discharge instructions are one area in which teach-back has been found to be particularly effective. Studies of methods to improve discharge instruction and decrease readmission rates have increased in number since a 2011 study that showed patients commonly remained confused about their condition, treatment, and discharge instructions after standard discharge [60]. Another study questioning patient comprehension and recall of discharge instructions showed that the majority of participants demonstrated an unsatisfactory level of comprehension regarding discharge information in at least one domain analyzed and that the majority of those were unaware of their own lack of understanding [61]. A meta-analysis by Oh et al. examined articles using teach-back education to confirm and reinforce patients’ comprehension of health-related information. They concluded that discharge directives delivered with the teach-back method resulted in a 45% reduction in 30-day readmissions. This study did have a high rate of selection bias due to limited trials, but the preliminary data support further inquiry [62].

Slater et al. in their 2017 study, added to the evidence when they showed that the teach-back method had a positive association on retention of discharge instructions in the emergency department regardless of age and education [63]. Despite growing evidence and teach-back being considered by some to be a “key discharge communication practice,” a 2021 survey of internal medicine residents revealed that only 17.0% of respondents reported routinely asking patients to “teach-back” or explain their understanding of the discharge plans. This study concluded that there is a disconnect between what we know to be best practice in discharge communication and what is actually demonstrated [23].

### Teach-Back in surgery

A literature search on teach-back in surgery yields limited results (Table 2). Pre-operative informed consent strategies showing either positive results or no change include written interventions [49, 68], audiovisual interventions [69], digital media interventions [70], and combinations of the above interventions [71]. A systematic review and meta-analysis comparing studies of these methods showed that interactive interventions, particularly with feedback or teach-back components, appear superior [64]. The available studies that utilized teach-back in surgical informed consent are summarized in Table 2.

**Table 2:** Results from trials of verbal discussion with test/feedback or teach-back interventions to improve patient comprehension in informed consent. These studies constitute the available literature on teach-back in surgical informed consent. Adapted from Glaser et al. 2020 [64]

Procedure	Intervention	Results	Reference
Spinal Stenosis Surgery	Routine, preoperative education followed by a “Knowledge Test Feedback Intervention”	Improved performance on knowledge test at admission, discharge, and at six months post-operation.	[65]
Carotid endarterectomy, laparoscopic cholecystectomy, radical prostatectomy, and total hip arthroplasty	Web-based tool with a knowledge check and a period for clarification before signing consent.	Total mean comprehension scores for all operations were 71.4% intervention vs. 68.2% control, *P* = 0.03 tested immediately after intervention	[66]
Various elective surgeries	A questionnaire was given immediately after informed consent with a teach-back component to assess time for a decision, satisfaction consent, and information provided about the proposed surgery (e.g., indications, benefits, risks, and alternatives).	Patients reported high satisfaction with teach-back during surgical informed consent. Teach-back is not detrimental to the consent process and may improve informed consent for surgery.	[67]

In a cohort of spinal stenosis surgical patients, Kesanen et al. provided routine preoperative education that included a face-to-face discussion with a surgeon and nurse, written material, and a “knowledge test feed-back intervention.” Patients were administered a 27-item true or false test, received the results with corrections, then completed an empowering telephone discussion with a nurse based on the patient’s existing knowledge. The patient’s understanding of risks, benefits, alternatives, and general knowledge about the procedure were assessed and compared with a control group who received only routine preoperative education. The intervention group showed superior performance on assessments at admission, discharge, and six months post-operation [65].

Fink et al., in a 2010 study on teach-back in carotid endarterectomy, laparoscopic cholecystectomy, radical prostatectomy, and total hip arthroplasty preoperative informed consent showed that the total mean comprehension scores for all operations after a 23-26 item questionnaire were 71.4% for the intervention group versus 68.2% for the control group. Standard informed consent was delivered using a web-based tool. When participants were ready to sign the consent, a teach-back dialog was initiated that prompted the provider to test the participant on essential information. The provider could then present additional information and instruction depending on the participant’s responses. The control group received standard informed consent using the same web-based tool with no additional support [66]. A follow-up to this study conducted by Prochanzka et al. in 2014 showed that surgical patients were highly satisfied with teach-back during the informed consent process, that teach-back did not deter from the process, and that teach-back improves informed consent [67].

## Conclusion

Teach-back is a valuable tool that can be employed to improve patient safety and understanding. It can provide the physician potent insight into the degree to which a patient understands the presented information. and can open a dialogue to resolve misunderstandings about the risks and benefits of a procedure. Unfortunately, the literature on the use of teach-back in the surgical informed consent process is minimal, despite evidence of its significant benefit in many other fields of medicine. However, some quality studies have established a baseline for advancing informed consent using methods such as teach-back. Improved patient knowledge benefits both the patient and the physician and enhances informed consent. Shared-decision making in the informed consent process may be enhanced by implementing teach-back in pre-operative discussions and its use should be further evaluated in the pre-operative informed consent process.

## Data Availability

All data and materials were obtained using public databases and have been cited appropriately.

## Abbreviation

AMA: American Medical Association
